# A streamlined method for analysing genome-wide DNA methylation patterns from low amounts of FFPE DNA

**DOI:** 10.1186/s12920-017-0290-1

**Published:** 2017-08-31

**Authors:** Jackie L. Ludgate, James Wright, Peter A. Stockwell, Ian M. Morison, Michael R. Eccles, Aniruddha Chatterjee

**Affiliations:** 1 Department of Pathology, Dunedin School of Medicine, University of Otago, 270 Great King Street, P.O. Box 56, Dunedin, 9054 New Zealand; 2 Maurice Wilkins Centre for Molecular Biodiscovery, Level 2, 3A Symonds Street, Auckland, New Zealand; 3 Department of Biochemistry, University of Otago, 710 Cumberland Street, Dunedin, 9054 New Zealand; 4 School of Biosciences, Cardiff University, Sir Martin Evans Building, Museum Avenue, Cardiff, CF10 3AX UK

**Keywords:** DNA methylation, DNA extraction, Bisulfite sequencing, RRBS, FFPE tissue, Genome-wide

## Abstract

**Background:**

Formalin fixed paraffin embedded (FFPE) tumor samples are a major source of DNA from patients in cancer research. However, FFPE is a challenging material to work with due to macromolecular fragmentation and nucleic acid crosslinking. FFPE tissue particularly possesses challenges for methylation analysis and for preparing sequencing-based libraries relying on bisulfite conversion. Successful bisulfite conversion is a key requirement for sequencing-based methylation analysis.

**Methods:**

Here we describe a complete and streamlined workflow for preparing next generation sequencing libraries for methylation analysis from FFPE tissues. This includes, counting cells from FFPE blocks and extracting DNA from FFPE slides, testing bisulfite conversion efficiency with a polymerase chain reaction (PCR) based test, preparing reduced representation bisulfite sequencing libraries and massively parallel sequencing.

**Results:**

The main features and advantages of this protocol are:

- An optimized method for extracting good quality DNA from FFPE tissues.
- An efficient bisulfite conversion and next generation sequencing library preparation protocol that uses 50 ng DNA from FFPE tissue.
- Incorporation of a PCR-based test to assess bisulfite conversion efficiency prior to sequencing.

**Conclusions:**

We provide a complete workflow and an integrated protocol for performing DNA methylation analysis at the genome-scale and we believe this will facilitate clinical epigenetic research that involves the use of FFPE tissue.

**Electronic supplementary material:**

The online version of this article (10.1186/s12920-017-0290-1) contains supplementary material, which is available to authorized users.

## Background

The development of next generation sequencing technologies has facilitated large-scale quantification of DNA methylation. The progressive improvement in profiling global DNA methylation provides a great opportunity for analyzing large numbers of clinical samples and detecting aberrant epigenetic marks [1]. Formalin-fixed, paraffin-embedded (FFPE) tissues represent a major source of samples in clinical research, especially in cancer research. In many cases, FFPE tissue is the only available material especially for retrospective studies. Therefore, it is essential to efficiently use FFPE tissues to obtain high resolution genomic and epigenomic data from clinical specimens.

However, FFPE is a challenging material for generating epigenomic data. The formalin fixation process leads to DNA damage due to fragmentation [2]. Indeed, nucleic acids from FFPE samples generally contain smaller fragments (less than 300 bp) [3, 4]. Further, the nature of tissue preparation leads to cross-linking of DNA and proteins [5]. The cross-linking process increases the mechanical stress on DNA and contributes to DNA degradation. In addition, non-buffered formalin that was used historically oxidizes to generate formic acid which results in DNA cleavage [6]. Further, several additional factors influence the quality of nucleic acids derived from FFPE; for example, duration of fixation, composition of fixative (concentration of formalin, pH and salt concentration), temperature and tissue type [3, 7–9]. Furthermore, processing of FFPE tissues for DNA extraction could affect the quality and downstream application. For good yield and quality, the lysis protocol that is used needs to effectively remove the DNA-protein cross links [10]. In addition, deparaffinization of the FFPE tissues is a crucial step. Deparaffinization procedures are considered to have great impact on the quality and quantity of nucleic acids extracted from FFPE blocks [11]. For DNA methylation analysis, bisulfite conversion is the most commonly used method that allows analysis of methylated and unmethylated CpG sites after the bisulfite treatment [12]. Bisulfite conversion will further degrade DNA [13, 14] and therefore methylation analysis on FFPE samples presents an additional challenge. Some previous studies have performed DNA methylation analysis of FFPE tissues [15–19]. These studies have provided valuable insights regarding the factors affecting methylation analysis of FFPE samples, and provided promising results for the use of FFPE material for DNA methylation profiling. However, in the previous sequencing-based methylation studies on FFPE samples, the mapping rates of sequenced reads (to the reference genome), were lower than those from fresh tissue.

**Fig. 1:**
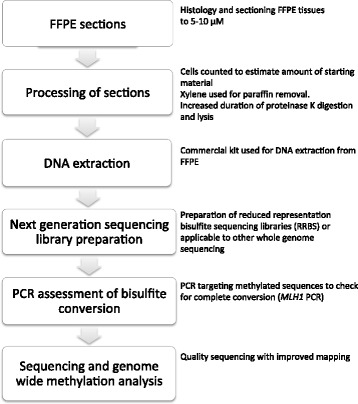
Diagram summarizing the workflow of experiments and key points for genome-wide DNA methylation analysis using FFPE samples

Here we describe a complete and optimized workflow for preparing next generation sequencing libraries for methylation analysis from FFPE tissues (Fig. 1). This includes, counting cells from FFPE blocks and extracting DNA from FFPE slides, testing bisulfite conversion efficiency with a polymerase chain reaction (PCR) based test and sequencing. We have optimized a method for extracting good quality DNA from FFPE tissues for methylation analysis. We have prepared next generation sequencing library (using reduced representation bisulfite sequencing [RRBS]) with 50 ng DNA from FFPE tissues and we demonstrate utility of a PCR-based test to assess bisulfite conversion efficiency prior to sequencing. Following the described protocol we obtained high quality methylation data and a higher mapping efficiency than previous studies.

## Methods

### Extraction of DNA from FFPE tissues

We have adopted the standard Qiagen kit protocol with some modifications. Additional incubation in proteinase K (steps 5 and 6) resulted in improved bisulfite conversion in some samples. The FFPE samples were obtained from primary, non-invasive (in-situ) melanomas. The FFPE blocks were collected under the regulation of Health and Disability Ethics Committee (Ethics protocol number: LRS1102002).

#### Materials

QIAamp DNA FFPE Tissue Kit (Qiagen #56404), with MinElute columns

Heating Block

Xylene (LabServ Analytical Grade #BSPXL864)

Proteinase K (Life Technologies #25530–015)

#### Procedure

1. FFPE tissue slices (10 μm thickness) were placed in 1.5 mL microcentrifuge tubes, 1 mL xylene was added and the tube was vortexed for 10 s. During DNA extraction from FFPE tissue, an important step is the efficient removal of paraffin as incomplete paraffin removal can inhibit further downstream applications such as PCR. We used a common technique for paraffin removal, which uses washes with xylene and ethanol [7]. We found that this technique worked very well with our FFPE samples. In our hands, using FFPE slices derived from the same blocks with a non-solvent based kit (Machery-Nagel Nucleospin FFPE DNA) did not give optimal DNA that could be amplified by PCR.
2. The tubes are then centrifuged for 2 min at 13000 rpm followed by removal of supernatant, leaving the pellet in the bottom of the tube.
3. Next, 1 mL 96% ethanol was added to each tube, and the tubes vortexed for 10 s followed by centrifugation at 13000 rpm for 2 min.
4. The top layer was removed and any remaining ethanol also removed with a fine pipette. The tube was then incubated in a heating block at 37 °C for 20 min (with the lid open) to evaporate the remaining ethanol.
5. The pellet was resuspended in 180 μL buffer ATL (from QIAmp DNA FFPE kit) with 20 μL proteinase K (20 mg/mL). The liquids were mixed by vortexing and the tube incubated in a heating block at 56 °C overnight.
6. After overnight digestion, an additional 100 μL ATL and 20 μL proteinase K was added and incubation at 56 °C was continued for at least two hours. Once the solution became clear (indicating complete lysis), the next step of DNA extraction then proceeded.
7. After lysis, clean-up steps were then performed as per the manufacturer’s protocol (Qiagen FFPE DNA Kit). Briefly, this involved incubation at 90 °C to reverse formaldehyde modification of DNA, treatment with RNase A, ethanol precipitation, binding of DNA to a MinElute (Qiagen) column and washing.
8. The DNA was finally eluted in 20 μL EB buffer and concentration measured using a NanoDrop (NanoDrop, USA).

### Assessment of DNA yields from the FFPE blocks

For extraction of nucleic acids from FFPE material, generally sections of 5–20 μm are used. However, the DNA yield from sections with the same thickness can vary substantially based on the number of cells present in a particular FFPE section. Excess starting material or cells can cause clogging of columns and could potentially result in inefficient extraction and poor yield. Performing a cell count on the desired FFPE section could provide an approximation of the expected DNA yield.

#### Materials

H & E stained 5 μm slides from FFPE blocks.

CS2 Aperio Digital Slide Scanner (Leica Biosystems)

Aperio Image Scope (Leica Biosystems) version: v12.2.2.5015 URL://www.leicabiosystems.com/digital-pathology/digital-pathology-management/imagescope/

Image J (National institutes of healthIH) version: 1.50i URL://imagej.nih.gov/ij/download.html

#### Procedure

1. FFPE tissues were cut into 5 μm thick sections, placed onto glass slides and stained with H&E.
2. Each slide was scanned using the Aperio Slide Scanner at 40X magnification.
3. Using Image Scope’s Pen Tool, for each FFPE sample (from step 3) several geographically distinct sections were defined (the number of defined sections varied between samples, ranging from 5 to 40). For each of these sections the area (in μm^2^ unit) was determined using Pen Tool.
4. Next, from each of these sections, a further 3 to 5 subsections were defined. The number of subsections measured from a section was determined by the total area size of section. Each subsection was recorded using the Snapshot tool at 40X magnification.
5. Using Image J, each subsection was then converted into an 8bit image with the background subtracted.
6. Next, we used threshold adjustment parameters on these subsections using Image J, this operation allows for the differentiation of cells from each other.
7. The image was then converted to “Mask”, followed by selection of “Fill Holes” option to fill any empty areas within each cell that was lost during threshold adjustment.
8. “Watershed” was then selected to provide a division point between the joint cells.
9. Next, we completed a particle analysis with a threshold pixel size of 120 and exclusion of cells from the edge of the sample.
10. Average cell count for a section was determined from the area and the number of cell in the subsections and then considering the total area of the section.
11. Because these calculations were done in a 5 μm section, each cell count was doubled to provide an approximate of the total cell count for 10 μm section.
12. The DNA yield reported here was measured using a Nanodrop (NanoDrop, USA). Nanodrop is widely available in standard molecular biology laboratories. For next generation sequencing applications, we recommend using the Qubit Fluorometer (Invitrogen), which provides sensitive assays for low amounts of DNA.

### Preparation of libraries for reduced representation bisulfite sequencing (RRBS) or other genome-wide methylation applications (*from FFPE samples)*

Following the extraction of DNA, the next step is to prepare libraries for sequencing to profile genome-wide methylation patterns. The genome-wide techniques employ a common principle for analysis; a local treatment of the genome to distinguish between methylated and unmethylated sites followed by global investigation of these modified sites to derive methylation patterns. The global investigation approaches are generally next generation sequencing or array platforms. For local treatment, there are three main approaches. These are: 1) restriction endonucleases that cleave DNA at specific recognition nucleotide sequences. 2) bisulfite conversion: treating DNA fragments with sodium bisulfite before PCR analysis. Sodium bisulfite treatment of DNA converts cytosine (C) residues to uracil (U), but leaves 5-methylcytosine residues unchanged [20]. RRBS falls under this category. One note is that sodium bisulfite treatment doesn’t distinguish between 5-methylcytosine and 5-hydroxymethylcytosine [21]. 3) an affinity enrichment method involving the application of an antibody (specific for methylated cytosines) to enrich for methylated regions in the genome by immunoprecipitating genomic DNA [22].

Sodium bisulfite treatment is a convenient and commonly used treatment prior to genome-wide sequencing. We used RRBS on FFPE samples to assess whether successful libraries could be prepared following the method described here. RRBS utilises bisulfite conversion combined with next-generation sequencing to provide single-nucleotide resolution methylation information at a genome-scale. Although we have tested the described protocol for RRBS, it is highly likely that these protocols could be used for other genome-wide methylation applications, as the principles are similar. The method for RRBS library preparation and its application has been extensively described previously [23–28] and therefore briefly described here, outlining modifications for working with FFPE derived DNA.

#### Materials (for RRBS library preparation from FFPE samples)

MspI restriction enzyme (NEB #R0106L)

Methylated adaptors

Illumina TruSeq Nano DNA LT Sample Prep Kit Set B (#FC-121-4002)

Zymo Research EZ DNA Methylation-Direct Kit (#D5021)

Agilent Technologies Pfu Turbo Cx Hotstart DNA polymerase (#600414)NuSieve GTG Agarose (Lonza #50080)

Invitrogen 25 bp DNA ladder (#10597–011)

Qiagen kits: MinElute PCR purification (#28006), MinElute gel extraction (#28604)

Thermocycler

2100 Agilent Bioanalyzer System

#### Procedure

1. Briefly, 50 to 500 ng genomic DNA extracted from FFPE material was digested overnight with MspI (a methylation insensitive restriction enzyme). A low starting amount of 50 ng resulted in a good PCR yield at the end of the protocol. It will be possible to further decrease the input DNA in future experiments.
2. The digested fragment was end-repaired, and 3′ A-overhang was added (using reagents from Illumina TruSeq Nano DNA LT Sample Prep Kit). Adding a 3′ A-overhang is required for ligating sequencing adaptors as these adaptors has a T overhang. Purification of the libraries was performed with MinElute PCR purification kit. Next, the methylated adaptors (Illumina, San Diego, CA) were ligated to the fragment. Following adaptor ligation, the libraries were bisulfite-converted with the EZ DNA methylation kit (Zymo Research, Irvine, CA #D5021). Next, a semi quantitative PCR (15 and 20 cycles) was performed to determine the minimum number of cycles required for large-scale amplification of the libraries (higher PCR cycle could introduce duplication bias). Remaining bisulfite-converted libraries were then amplified at large-scale by PCR (between 15 to 20 amplification cycle).
3. After successful amplification, 150 to 330 bp fragments (post-adaptor ligation size; this corresponds to 40–220 bp fragments pre-adaptor ligation) were size-selected from 3% Nusieve agarose gels (Lonza, Basel, Switzerland). A modification compared to the previous protocols is that we have performed size selection at the end of library preparation (i.e., after bisulfite conversion and PCR amplification). This modification allows us to use a low amount (e.g. 50 ng) of input DNA. Note that we have performed size-selection (of 40–220 bp) as per the original RRBS protocol [29]; however, it is also possible to select any other size ranges for reduced representation libraries.

### Assessment of bisulfite conversion efficiency with PCR before next generation sequencing

Next-generation sequencing of the prepared libraries is an expensive step. The most critical aspect for the success of methylation-sequencing libraries is efficient bisulfite conversion. Therefore using commercially available kits, we have incorporated a PCR based test for assessing the success of bisulfite conversion for methylation-sequencing libraries. Here we describe this method and also demonstrate the utility of this test with an example from generating FFPE RRBS libraries.

#### Materials

Zymo Universal Methylated Human DNA Standard and Control Primers (cat # D5011.)

Zymo Taq PreMix (cat # E2003)

### RRBS library sequencing:

Sequencing of the RRBS libraries were performed using Illumina MiSeq machine. Single-ended, single-ended 151 bp reads were obtained for downstream analysis.

#### Procedure

1. Using the Zymo Universal Methylated Human DNA Standard and Control Primers (cat # D5011) as a positive control we tested bisulfite conversion efficiency before next generation sequencing.
2. This product contains DNA in which all the cytosines in CpG dinucleotides have been enzymatically methylated using M.SssI methyltransferase (referred as Methylated +ve control). The methylated cytosines in CG dinucleotides remain unconverted following bisulfite treatment, where unmethylated Cs are converted into uracil and detected as thymine after PCR. If the conversion is successful (i.e., all the non CpG cytosines are converted and detected as thymine), the primers are able to bind and a PCR product can be seen in the diagnostic agarose gel. The methylated CG sequence in the control DNA provides additional specificity for primer binding. If the control DNA does not show any product after PCR, it is likely to indicate poor bisulfite conversion.
3. Bisulfite conversion was performed on the desired samples (i.e., adaptor ligated RRBS libraries according to the current protocol) along with the Methylated +ve control (50 ng DNA in 20 μL as per manufacturer’s instructions).
4. A bisulfite PCR with the Zymo control primers was then performed on the desired samples and the control DNA to test the efficiency of conversion. This PCR is designed to amplify a 182 bp fragment of the human *MLH1* mismatch repair gene after successful bisulfite treatment. This method uses only *MLH1* gene as a control; however, it is possible to incorporate a panel of multiple genes for testing bisulfite conversion.
5. Details of the PCR primers:

*MLH1* Primer I:

5′ - GGAGTGAAGGAGGTTACGGGTAAGT - 3′

*MLH1* Primer II:

5′ - AAAAACGATAAAACCCTATACCTAATCTATC - 3′

PCR mix: (Zymo Taq Premix (#E2003)

12.5 μL 2X Zymo Taq PreMix

1.0 μL primer mix (contains both hMLH primers)

2.0 μL bisulfite treated DNA

9.5 μL dH_2_0

Final volume = 25 μL (23 μL mix +2 μL bisulfite converted DNA).

6. The primer annealing temperature was 59 °C and 35 cycles of PCR were performed. The product was run on a 2% agarose gel, stained with ethidium bromide and visualised under UV light. The presence of a 182 bp band shows that conversion is successful.

## Results

### Assessment of DNA yields from FFPE

**Fig. 2:**
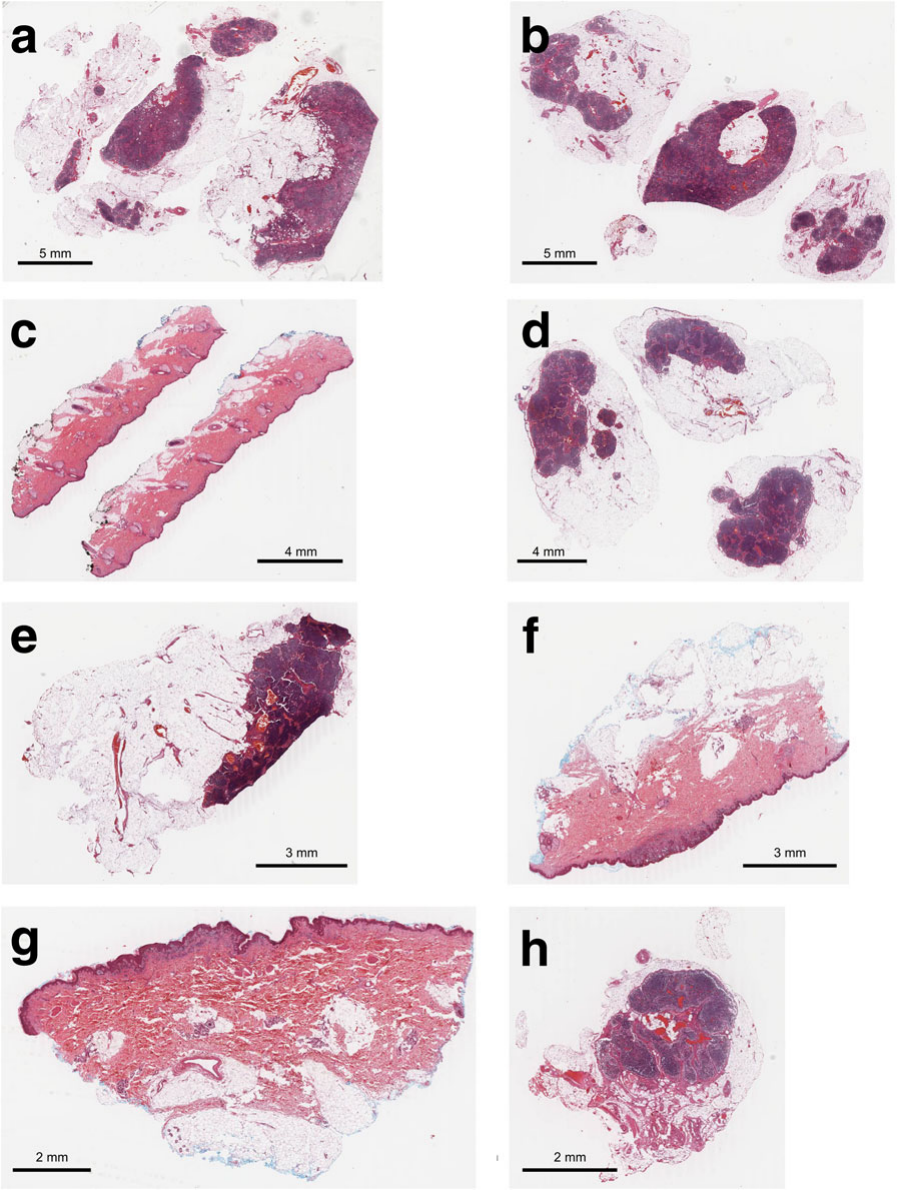
H&E stained 5 μm cut sections from formalin fixed paraffin embedded (FFPE) melanoma. Eight analysed FFPE samples (**a**–**h**) are shown. Scales bars are shown

We performed cell counting of eight FFPE sections and compared the DNA yield (Fig. 2 and Figure S1 in Additional file 1). We counted the cell number in 5 μm sections and doubled the cell count to provide an approximation of the total cell count for 10 μm section (results are shown in Table 1). We confirmed that the number of cells in a 10 μm section was strongly correlated with the total DNA yield from that section (Pearson *r* = 0.67, correlation after log2 transformation = 0.74, Figure S2 in Additional file 1). However, the observed and expected recovery rates substantially differed between samples. Here we provide the method that we used for counting the number of cells in a 10 μm FFPE slide and the corresponding DNA yield for these samples (Table 1). However, this represents one of many possible methods for counting cells and measuring DNA yield. The calculated total cell counts should serve as an estimate, as these are determined on an average of the representative sub-sections within an FFPE slide.

**Table 1:** Number of cells in an FFPE section and corresponding DNA yield

Sample	Number of cells	DNA yield (ng)	260/280	Size of FFPE sections
A	2.0 × 10^6^	7760	1.86	30 × 25
B	1.9 × 10^6^	6110	1.82	30 × 25
C	1.6 × 10^5^	2060	1.59	25 × 25
D	2.3 × 10^6^	2565	1.8	30 × 25
E	4.1 × 10^5^	1435	1.78	25 × 25
F	6.5 × 10^4^	340	1.48	25 × 25
G	1.1 × 10^5^	405	1.77	25 × 25
H	1.4 × 10^5^	4150	1.51	15 × 15

### Preparation of libraries for reduced representation bisulfite sequencing (RRBS) or other genome-wide methylation applications (*from FFPE samples)*

**Fig. 3:**
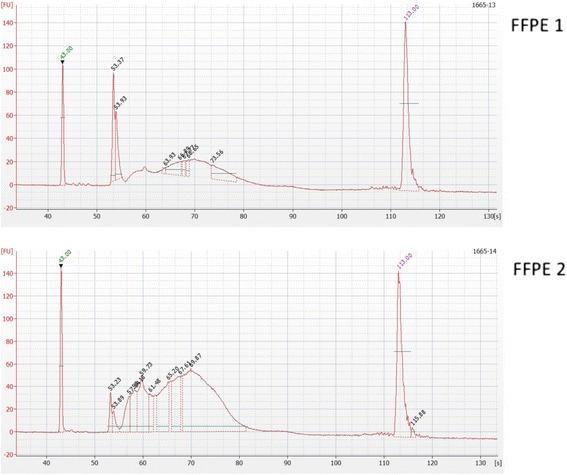
Bioanalyser images demonstrating quality of two FFPE RRBS libraries. Each of the RRBS libraries (FFPE1 and FFPE2) was run on an Agilent 2100 Bioanalyzer using the high sensitivity DNA kit. The electropherogram displays a plot of fragment size (bp) versus fluorescence intensity. Peaks at 35 bp and 10,380 bp represent lower and upper markers. The 160–340 bp peaks represent the RRBS library

The quality of the RRBS libraries prepared from melanoma FFPE slices was assessed on a 2100 Bioanalyzer (Agilent Technologies) using the high sensitivity DNA chip. Bioanalyzer analysis of two representative FFPE derived RRBS libraries are shown in Fig. 3.

### Assessment of bisulfite conversion efficiency with PCR before next generation sequencing

**Fig. 4:**
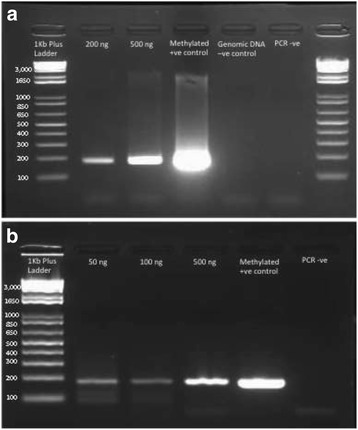
*MLH1* PCR to test efficiency of bisulfite conversion on FFPE derived DNA. **a**) *MLH1* PCR of FFPE-derived bisulfite-treated DNA. Lane 1: 1Kb + ladder, Lane 2: 200 ng input DNA, Lane 3: 500 ng input DNA, Lane 4: Zymo methylated control DNA, Lane 5: unconverted genomic DNA, Lane 6: PCR negative (water). 2% agarose, run for 25 mins at 100 V. **b**) *MLH1* PCR of RRBS libraries prepared from different amounts of FFPE-derived DNA. FFPE DNA was digested with MspI enzyme, A-tailed, end repaired and ligated to Illumina adaptors, and bisulfite converted. Then PCR was performed with *MLH1* primers. Lane 1: 1Kb + ladder, Lane 2: 50 ng input DNA, Lane 3: 100 ng input DNA, Lane 4: 500 ng input DNA, Lane 5: Zymo methylated control DNA, Lane 6: PCR negative (water). 2% agarose, run for 25 mins at 100 V

Figure 4a shows an example of the *MLH1* PCR performed on bisulfite-converted FFPE-derived DNA and the control DNA. The DNA concentration (200 and 500 ng, lane 2 and 3 in Fig. 4a) is the amount of DNA added to the initial bisulfite reaction, with 2 μL of bisulfite DNA used per PCR. Zymo Universal methylated human DNA standard (lane 4, methylated +ve control) was used as a positive control as described above. This test was performed on FFPE DNA samples without preparing RRBS library. As this test could be used to test bisulfite conversion for any DNA methylation application, we have shown this image to demonstrate the success of this protocol with library preparation. The FFPE DNA samples and control DNA show a band at 182 bp as expected suggesting all these samples were successfully converted. In addition, as expected, the untreated genomic DNA and PCR blank (dH_2_0) showed no amplification (lanes 5 and 6 respectively).

We have shown the results for this PCR test for RRBS libraries in Fig. 4b. RRBS libraries were prepared with different input DNA and then bisulfite converted and amplified with Illumina primers to recover enough DNA for next generation sequencing (lane 2–4 contains libraries prepared with 50, 100 and 500 ng DNA input respectively, lane 5 contains a methylated positive control and lane 6 contains a water negative control, Fig. 4b). Similar to previous observations a 182 bp was visible in the gel as expected suggesting that all these libraries were successfully converted. We also noticed an additional band at ~120 bp (Fig. 4b). We have previously described the presence of this additional band which is likely to arise from adaptor-adaptor dimerization [30]. For all three libraries (50, 100 and 500 ng input), the same adaptor concentration was used and the gel image indicates that higher intensity of potential adaptor dimers in libraries with lower DNA templates (i.e., a stronger band from the 50 ng library compared to the 500 ng library, Fig. 4b).

### Sequencing, alignment and analysis of RRBS libraries from the FFPE DNA samples

To test whether good quality RRBS sequences could be obtained from the FFPE libraries, we performed massively parallel sequencing on two FFPE RRBS libraries (FFPE1 and FFPE2 as shown in Figs. 3 and 4). For this test purpose, Illumina MiSeq was used to generate single-ended 151 bp sequences. We obtained 109,352 and 78,364 sequenced reads for FFPE1 and FFPE2 samples respectively. We assessed the quality of the sequenced reads by plotting Phred quality scores along the read position using the FastQC program (from Babraham Institute, URL://www.bioinformatics.babraham.ac.uk/projects/fastqc/). The higher the Phred score, the better was the base call (calculated using the formula Qphred = −10 log10(e), where e is the estimated probability of a base being incorrectly identified). For both the FFPE libraries, very high quality sequenced reads were obtained (mean quality score of 32 and 35 for FFPE1 and FFPE2 respectively, Figure S3 in Additional file 1). Consistent with this observation, we also found no traces of N bases (i.e., if the base-caller cannot determine the sequence, it replaces these bases with Ns which are not usable and cause misalignment if present in the dataset) in both of these samples (Figure S4 in Additional file 1). The Illumina platform uses sequence by synthesis chemistry to sequence the DNA molecules and as a result of accumulation of errors, the base calling is less accurate at the end of the reads (3′ end). Our sequenced reads were 151 bp long and we observed relatively decreased sequence quality towards the 3′ end of the sequence, consistent with previous quality reports on RRBS sequenced reads (Figure S3 in Additional file 1).

Finally, we aligned these sequenced reads with the reference human genome (GRCh37 build) using bisulfite aligner Bismark (version: v0.14.3). Alignment was performed after processing the reads and removal of adaptors as described previously [20, 31]. After processing, 63,870 and 69,988 sequenced reads were accepted for analysis and alignment for FFPE 1 and FFPE 2 respectively. This was an acceptance rate of 58.4% and 89.3% sequenced reads for further analysis. In the human genome, especially in differentiated somatic cells the proportion of non-CpG methylation is low [32, 33]. If an RRBS or other whole genome-scale bisulfite treated library shows a high level of non-CpG methylation, it is likely that this results stem from failure of bisulfite conversion. The Bismark analysis of FFPE1 and FFPE2 samples indicated a very low level of non-CpG methylation. For FFPE1, both the CHG and CHH context methylation was 1.2%, while for the FFPE2 library, CHG and CHH context methylation was 1.2% and 1.1% respectively (Data S1 and S2 in Additional file 1). This percentage is the summation of the actual non-CpG methylation in the genome and incomplete bisulfite conversion. Taken together, these results indicate a high efficiency of bisulfite conversion in these FFPE libraries, consistent with the PCR test described in Fig. 4 for the several FFPE samples. Recently, unmethylated Lambda DNA spike-in was used to test bisulfite conversion efficiency in RRBS libraries. After sequencing and alignment, the non-conversion rate was calculated as the number of sequenced cytosines in non-CG contexts divided by all the covered cytosines in non-CG contexts in the lambda DNA genome [34].

## Discussion

For retrospective clinical studies and archival biological material, FFPE represents the most common tissue resource. The ability to perform epigenetic analysis will not only help in understanding the molecular basis of diseases but also has implications for other ongoing work involving the development of new epigenetic biomarkers or diagnostic assays. Previously, few studies have assessed the quality of DNA methylation profiles obtained from FFPE tissues [35–37]. These studies have reported a good correlation of methylation calls between fresh tissues and FFPE samples. Also, it was shown that results obtained from FFPE tissues were reproducible with independent techniques for methylation profiling. However, these analyses were based on few CpG sites and mainly captured the promoter methylation status of selected genes. Here we provide a complete workflow and protocol for performing genome-wide methylation analysis and highlight critical factors for successful analysis of FFPE samples (see Table S1 in Additional file 1). We also implemented and demonstrated a PCR based method to assess bisulfite conversion efficiency prior to sequencing. This could be used to screen samples prior to sequencing so that only successfully converted samples would go to the next step, leading to significant savings in cost and time.

Due to DNA degradation and fragmentation, the mapping rates for FFPE samples are lower than those that would be expected from fresh tissue or cell line material. For example, a previous genome-wide evaluation of FFPE material reported unique mapping rates of 7.0% to 19.9% [16]. Following the protocol described here, we obtained a unique mapping efficiency of 35–40% (Data S1 and S2 in Additional file 1). This mapping efficiency was obtained with a stringent mapping criteria of only one mismatch in the seed of the sequenced read (i.e., in the first 28 bp of the reads) while the default mismatch allowed in Bismark alignment is two. If the default parameters are used the mapping efficiency is likely to further improve. Furthermore, a previous study which sequenced 18 FFPE samples using RRBS, reported unique alignment rate of the sequenced reads ranging from 16.7% to 53.1% (median = 27.7%) [15]. Following the described protocol we obtained 58.4% and 89.3% reads that passed quality control and were used for alignment to the reference genome.

## Conclusions

As a method for genome-wide methylation profiling, RRBS is shown to be reproducible and has been widely used by many groups world-wide [15, 38–43]. In a recent analysis of melanoma cell lines, we further demonstrated reproducibility of RRBS results for several target genes, using Sequenom EpiTyper methylation analysis and traditional bisulfite sequencing [44, 45]. Here we have combined several methods to provide an integrated protocol. In the current study, we have not directly compared our FFPE RRBS pipeline with a complete existing pipeline. Future comparison of the genome-wide methylation profiles of FFPE samples using the described RRBS workflow with other global methylation analysis will be beneficial to further demonstrate the utility of this method. However, we have demonstrated a cell counting method that optimises tissue usage when analysing small samples (for example, primary melanoma). We have shown successful bisulfite conversion of genomic DNA extracted from an extended proteinase K treatment of FFPE tissue, whereas standard DNA extraction protocols resulted in bisulfite conversion failure in some libraries. In addition, we have successfully implemented a PCR-based method to assess bisulfite conversion before and after RRBS library preparation that avoids sequencing of poor quality libraries. Using this integrated protocol, we have demonstrated better mapping efficiency than previously published genome-wide methylation studies. In conclusion, we provide a streamlined workflow and protocol for performing DNA methylation analysis at the genome-scale and we believe this will facilitate clinical epigenetic research that involves the use of FFPE tissue.

## Additional file

Additional file 1: Supplementary information. (DOCX 226 kb)

## Abbreviations

FFPE: Formalin fixed, paraffin embedded
PCR: Polymerase chain reaction
RRBS: Reduced representation bisulfite sequencing
