# Supplementary material for: A streamlined method for analysing genome-wide DNA methylation patterns from low amounts of FFPE DNA
Source: BMC Med Genomics. 2017 Aug 31;10:54. doi: 10.1186/s12920-017-0290-1 (PMC5580311; doi:10.1186/s12920-017-0290-1)
Supplement: Additional file 1: — Supplementary information. (DOCX 226 kb) [file 12920_2017_290_MOESM1_ESM.docx]

**Additional file 1 for**

**An optimised method for analysing genome-wide DNA methylation patterns from low amounts of FFPE DNA**

**Jackie L Ludgate^1^, James Wright^1,4^, Peter A Stockwell^3^, Ian M Morison^1^, Michael R Eccles^1,2^, Aniruddha Chatterjee^1,2*^**

^1^Department of Pathology, Dunedin School of Medicine, University of Otago, 270 Great King Street, Dunedin 9054, New Zealand. ^2^Maurice Wilkins Centre for Molecular Biodiscovery, Level 2, 3A Symonds Street, Auckland, New Zealand. ^3^Department of Biochemistry, University of Otago, 710 Cumberland Street, Dunedin 9054, New Zealand. ^4^School Of Biosciences, Cardiff University, Sir Martin Evans Building, Museum Avenue, Cardiff, CF10 3AX, United Kingdom.

*To whom correspondence should be addressed: Aniruddha Chatterjee, Department of Pathology, Dunedin School of Medicine, University of Otago, P.O. Box 56, Dunedin, New Zealand, Telephone: +64 3 470 3455, E-mail: aniruddha.chatterjee@otago.ac.nz

**Includes:**

**Supplementary Figures S1-S4**

**Supplementary Table S1**

**Supplementary Data S1 and S2**

**Supplementary Figures**

**
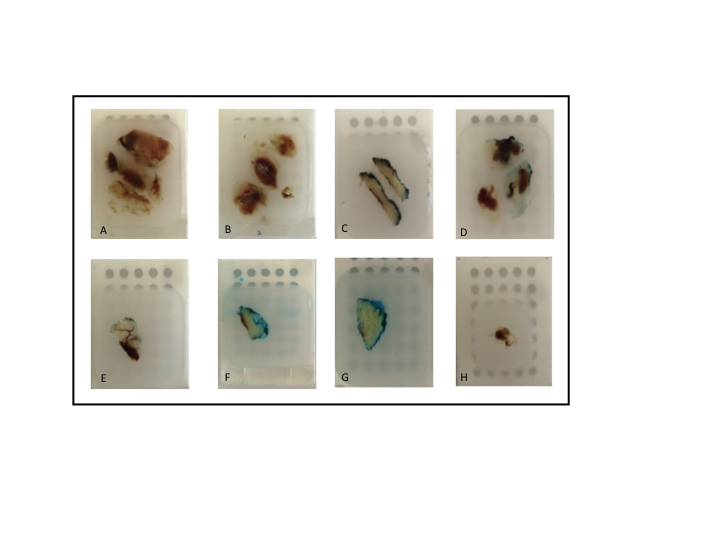
**

**Figure S1: Images of the actual eight FFPE blocks analysed for DNA extraction in the current study.**

**Figure S2: Relationship of number of cells counted in each FFPE block analysed and the corresponding DNA yield.** The red line represents regression line (y~x).

**
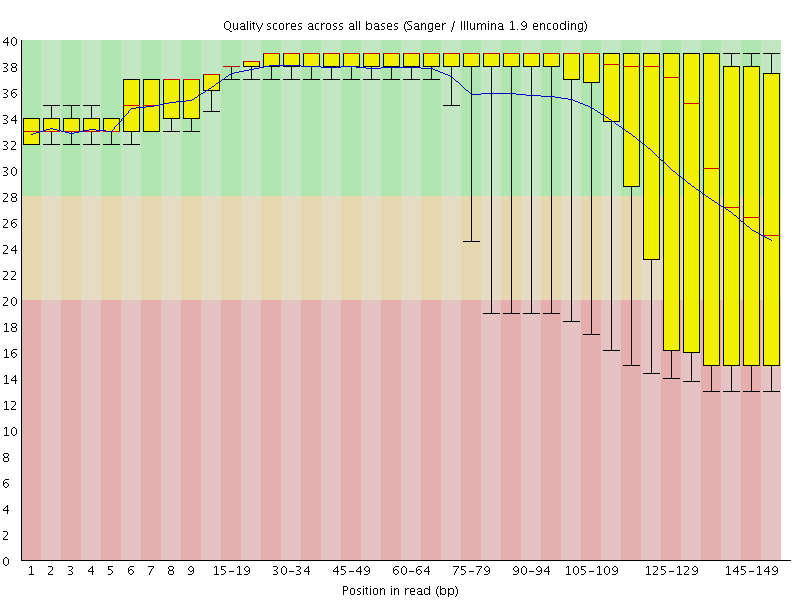
**

**Figure S3: Sequence quality as indicated by FastQC for FFPE2.** For each set of positions a BoxWhisker type plot is shown. The central red line is the median. The yellow box represents the inter-quartile range (25-75%). The upper and lower whiskers represent the 10% and 90% points. The blue line shows the mean quality. The Y-axis shows the Phred quality scores.


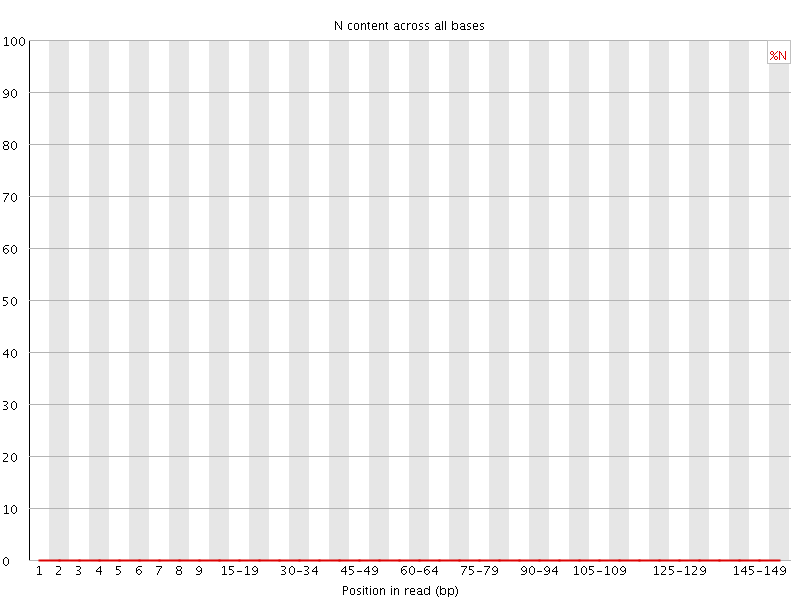


**Figure S4: Per base N content as indicated by FastQC for FFPE2.** The X-axis shows the sequencing cycle or positions in reads. The Y-axis shows percentages of occurrence of N along the read.

**Supplementary Table**

**Table S1: A summary of the important factors to consider while performing genome-wide methylation analysis on FFPE tissues.**

| **Steps** | **Factors to consider** |
| --- | --- |
| FFPE block | Estimate the number of cells in the block to get a prior estimate of DNA yield |
| DNA extraction | Effective deparaffinization |
|  | Extended protease treatment of DNA |
| Bisulfite conversion | Assess conversion efficiency with PCR test. A multi-gene panel PCR test could be used |
|  | Add lambda DNA to test conversion efficiency post sequencing |
| PCR | Carefully evaluate the PCR gel to determine the optimal PCR cycle to amplify the library and provide enough DNA for sequencing. |
| Data processing | Assess quality using FASTQC and other methods |
| Mapping | Evaluate different mapping parameters (for example, number of mismatches to be allowed while mapping) to maximise mapping rates. |

**Data S1: RRBS on sample FFPE #1 (13) – adaptor trimmed, from MiSeq run**

Bismark report for: ./FFPE1_R1_adtr.fastq (version: v0.14.3)

Option '--directional' specified (default mode): alignments to complementary strands (CTOT, CTOB) were ignored (i.e. not performed)

Bismark was run with Bowtie against the bisulfite genome of /Volumes/Data2/HomoSapiens_genome/hs_ref_GRCh37/ with the specified options: -q -n 1 -k 2 --best --chunkmbs 512

Final Alignment report

======================

Sequences analysed in total: 63870

Number of alignments with a unique best hit from the different alignments: 22296

Mapping efficiency: 34.9%

Sequences with no alignments under any condition: 35056

Sequences did not map uniquely: 6518

Sequences which were discarded because genomic sequence could not be extracted: 0

Number of sequences with unique best (first) alignment came from the bowtie output:

CT/CT: 11257 ((converted) top strand)

CT/GA: 11039 ((converted) bottom strand)

GA/CT: 0 (complementary to (converted) top strand)

GA/GA: 0 (complementary to (converted) bottom strand)

Number of alignments to (merely theoretical) complementary strands being rejected in total: 0

Final Cytosine Methylation Report

=================================

Total number of C's analysed: 373468

Total methylated C's in CpG context: 23834

Total methylated C's in CHG context: 1281

Total methylated C's in CHH context: 2405

Total unmethylated C's in CpG context: 44787

Total unmethylated C's in CHG context: 101911

Total unmethylated C's in CHH context: 199250

C methylated in CpG context: 34.7%

C methylated in CHG context: 1.2%

C methylated in CHH context: 1.2%

**Data S2: FFPE #2 (14) – adaptor trimmed, from MiSeq run**

Bismark report for: ./FFPE2_R1_adtr.fastq (version: v0.14.3)

Option '--directional' specified (default mode): alignments to complementary strands (CTOT, CTOB) were ignored (i.e. not performed)

Bismark was run with Bowtie against the bisulfite genome of /Volumes/Data2/HomoSapiens_genome/hs_ref_GRCh37/ with the specified options: -q -n 1 -k 2 --best --chunkmbs 512

Final Alignment report

======================

Sequences analysed in total: 69988

Number of alignments with a unique best hit from the different alignments: 27972

Mapping efficiency: 40.0%

Sequences with no alignments under any condition: 33643

Sequences did not map uniquely: 8373

Sequences which were discarded because genomic sequence could not be extracted: 0

Number of sequences with unique best (first) alignment came from the bowtie output:

CT/CT: 13860 ((converted) top strand)

CT/GA: 14112 ((converted) bottom strand)

GA/CT: 0 (complementary to (converted) top strand)

GA/GA: 0 (complementary to (converted) bottom strand)

Number of alignments to (merely theoretical) complementary strands being rejected in total: 0

Final Cytosine Methylation Report

=================================

Total number of C's analysed: 475494

Total methylated C's in CpG context: 28996

Total methylated C's in CHG context: 1549

Total methylated C's in CHH context: 2760

Total unmethylated C's in CpG context: 63516

Total unmethylated C's in CHG context: 128899

Total unmethylated C's in CHH context: 249774

C methylated in CpG context: 31.3%

C methylated in CHG context: 1.2%

C methylated in CHH context: 1.1%
